# Promoting Foundation Reading Skills With At-Risk Students

**DOI:** 10.3389/fpsyg.2021.671733

**Published:** 2021-07-05

**Authors:** Ana Sucena, Ana Filipa Silva, Cátia Marques

**Affiliations:** Research and Intervention Reading Centre, Polytechnic Institute of Porto, Porto, Portugal

**Keywords:** assessment, early reading program, letter-sound knowledge, phonemic awareness, decoding

## Abstract

This paper presents an early reading intervention program, the PPCL (*Programa de Promoção das Competências Leitoras*—Promoting Reading Skills Program). PPCL focuses on the promotion of reading foundation abilities—letter-sound, phonemic awareness, decoding, and spelling—with at-risk first graders. This study assessed the impact of PPCL on the reading foundation abilities with 311 first graders (173 boys and 138 girls), divided between intervention and comparative group (respectively, 206 and 105 first graders). Results were analyzed with an inter- (intervention and comparative group) and intra- (pre-and post-test) group design. A mixed two-way Manova indicated the presence of statistically significant differences between the two assessment moments, with the intervention group presenting higher values than the comparative group in all abilities at the post-test and also above the cutoff score in all variables, which indicates that at-risk students eventually concluded the school year with satisfactory levels of reading skills. On the other hand, the comparative group scored below the cutoff score in all variables. The magnitude of the effect on the intervention group was higher than the one observed in the comparative group. Reading promotion with PPCL significantly improved at-risk students reading skills. In future studies, the authors intend to follow up on reading and writing participants’ skills.

## Introduction

Reading and writing are indispensable skills, of crucial importance for equal opportunities (Blomert and Froyen, 2010). Impairments in reading and writing acquisition skills can seriously limit personal aspirations (Jeffes, 2016; Jamshidifarsani et al., 2019). Reading and writing acquisition are based on the learning rhythm, which is associated with the characteristics of the orthography in which the child acquires reading. The differences between orthographies regarding their consistency between graphemes and phonemes are the basis for the Orthographic Depth Continuum. The less transparent an orthography is, the greater the likelihood for children to experience difficulties, especially in the initial phase of reading acquisition. European Portuguese is considered an intermediate orthography thus allowing children to develop the reading foundations at a faster pace and experiencing fewer difficulties than English children for example (Sucena et al., 2009; Ziegler et al., 2010). English is an opaque orthography, with 44 phonemes (with more than 1,100 possible pronunciations) and 229 graphemes (Rao, 2018). As a result of the inconsistencies between graphemes and phonemes English speaking children need a longer learning period (two times longer) to acquire the reading foundations (Seymour et al., 2003). Unlike English, Portuguese orthography presents fewer inconsistencies between graphemes and phonemes—35 phonemes for 67 graphemes (Serrano et al., 2011). Even if Portuguese speakers acquire reading skills earlier and faster than English speakers, some Portuguese children still face difficulties and struggle to develop reading and spelling skills.

For reading acquisition to be possible, two fundamental basic skills must be developed: phonemic awareness and letter-sound knowledge (Seymour and Evans, 1999; Seymour et al., 2003; Sucena et al., 2009, 2015; Kyle et al., 2013; Viana and Sucena, 2014). Phonemic awareness, the ability to analyze sounds that constitute words, is crucial for reading acquisition and considered one of the most powerful predictors of reading and writing success (Snowling, 2014; Landerl et al., 2019). It facilitates the understanding of correspondences between phonemes and the corresponding letters (Hulme et al., 2012). Difficulties with phonemic awareness are more commonly found across students who enter the school from socially disadvantaged backgrounds (Zalewska-Łunkiewicz et al., 2016; Diuk et al., 2019), who have experienced difficulties with language development (Bickford-Smith et al., 2005; Porta and Ramirez, 2019; Raspin et al., 2019), and/or at family risk of dyslexia (Pennington and Lefly, 2001). Research has also shown that training phonemic awareness results in significant gains in reading and spelling performance. In the same vein, the performance of spelling and decoding are strongly related to phonemic awareness and letter-sound knowledge (Taha and Saiegh-Haddad, 2015). When children enter the first grade with low levels of phonemic awareness and letter-sound knowledge there is an increased risk for reading and writing difficulties. It is thus of major importance to identify them as being at risk of developing learning difficulties and beginning a systematic intervention.

These findings provide the theoretical motivation for early reading intervention programs (Hatcher et al., 2006; Suggate, 2016). The most successful reading intervention programs combine phonological awareness training with structured reading explicit instruction (e.g., Hatcher et al., 1994; Lovett et al., 2003; Hatcher et al., 2006; Snowling and Hulme, 2011; Galuschka et al., 2019) using self-correction strategies (Williams et al., 2016). A meta-analysis assessment of the long-term effects of phonemic awareness shows that the impact of phonemic awareness interventions was maintained along the time and transferred to non-targeted skills such as letter-sound knowledge with consequences on reading comprehension (Suggate, 2016).

Interventions with the best results in reading skills are those occurring early, i.e., in kindergarten (Hatcher et al., 2004; Richardson and Lyytinen, 2014) or at the beginning of primary school (with 6 or 7-years old) (Wimmer and Mayringer, 2002; Saine et al., 2011). If reading disabilities are not addressed early with at-risk students, difficulties tend to generalize to other domains thus jeopardizing future knowledge acquisition (Raspin et al., 2019), exposing students to consecutive experiences of failure, thereby diminishing their motivation to learn (Lyytinen and Erskine, 2016). However, when these difficulties are identified early and lead to a prompt individualized or small group systematic and intensive intervention, the likelihood of reversing failure trajectories is very high (Lyytinen, 2008; Hall and Burns, 2018).

Such findings have in recent years led to recommendations for reading instruction. The OECD (Organisation for Economic Co-operation and Development) formulated recommendations to help constituent countries to find solutions that enable effective learning. In Portugal, the report of Rodrigues et al. (2017) recommends early and continuous intervention for reading and writing teaching, during the initial years of school, i.e., from 1st to 4th grade. It also recommends the development of diagnostic and intervention tools and the sharing of information resources, early diagnosis, and intervention methodologies to be used in conjunction with teaching strategies and individualized intervention by teachers (Rodrigues et al., 2017).

The implementation of such policies tends to provide students with solid reading foundational abilities, such as phonemic awareness and letter-sound knowledge at school entry. Research suggests the existence of a strong cause-effect relationship between children’s prior knowledge (including phonological awareness and letter-knowledge) and success in reading and writing acquisition at first grade (Birgisdottir et al., 2020). It is well known that the way children begin formal schooling influences their school path (Stanovich, 1991). There is a tendency for those who begin the journey without difficulty to build successful paths. Conversely, for those whose beginning is marked by difficulties (at-risk students), there is a tendency for an increase in difficulties throughout schooling, with consequences on the motivation toward school and school learning (Lyytinen and Erskine, 2016). In this sense, the preparation of students for the reading acquisition should include phonemic awareness and letter-sound knowledge, especially so at the onset of first grade and preferably also at kindergarten.

In Portugal, more than 10% of children are retained in the 2nd grade mainly because of reading difficulties (e.g., Ferreira et al., 2015). This scenario is also found in other countries such as Luxemburg, Netherland, France and Spain (PISA, 2012). We can assume that the mainstream educational system still fails to support students at risk of developing learning disabilities. The opportunities usually given to students with learning disabilities are not enough for them to consolidate letter-sound knowledge, to develop effective spelling, decoding and reading fluency (Schaars et al., 2017; Duke and Mesmer, 2019). In this sense, international research has invested in the development and assessment of the impact of intervention projects that inform the educational practice, promoting the educational success of first graders (Niedo et al., 2014; López-escribano et al., 2014; Nagler et al., 2015; Shannon et al., 2015; Snellings et al., 2015; Alber-Morgan et al., 2016; Ardoin et al., 2016; Hollands et al., 2016; Schneider et al., 2016; Horne, 2017; Madden and Slavin, 2017; Messer and Nash, 2017; Moser et al., 2017; Rosas et al., 2017; van de Ven et al., 2017; Solheim et al., 2018; Jamshidifarsani et al., 2019; Martens et al., 2019; Volkmer et al., 2019).

These studies show that successful reading interventions focus primarily on four major topics: phonemic awareness, decoding, fluency, and comprehension. Studies that used a comparative group revealed differences between the intervention and non-intervention groups, with more marked improvements in reading skills for the intervention group (e.g., Nagler et al., 2015; Horne, 2017; Messer and Nash, 2017; Rosas et al., 2017). Similar initiatives have been developed in Portugal. A report of PNPSE (Portuguese acronym for Programa Nacional de Promoção do Sucesso Escolar, in English National School Success Promotion Program) published in 2019, describes the projects implemented in Portuguese primary schools between 2016 and 2018 (in Portugal, the primary school system includes the 1st to the 4th grade). The report lists six projects on reading and writing acquisition (Verdasca et al., 2019). These projects focus on different reading competencies. Three of these projects focus on fluency and comprehension, one on phonemic awareness, letter-sound knowledge and decoding; no specific information is presented for the remaining. Three main axes can be highlighted from these projects: (i) tracking and monitoring (ii) consultancy, and (iii) intervention. Projects focusing on tracking and monitoring aim to track and monitor the students reading fluency and comprehension, by analyzing the students’ performance during the school year. Projects focusing on consultancy aim to train the technical team, responsible for implementing the project, on the mastery of intervention strategies and follow-up the efficacy of their interventions. Finally, the intervention was the main goal of one of these projects, focusing on developing and implementing intervention strategies on phonemic awareness and letter-sound knowledge development, as well as the decoding process with speech therapists, psychologists and school teachers working full time in the project. This last project is the subject of analysis in the present study. As far as the authors’ knowledge goes, this is the first study to present the intervention impact (using a comparative and intervention group) for a Portuguese reading promotion program.

### The PPCL

This intervention program is part of a broader project, aiming not only at at-risk first graders but also kindergarteners. In this study, we focus on the at-risk first graders’ intervention program. This program aims to intervene early with at-risk students promoting the alphabetic principle learning through phonemic awareness and letter-sound knowledge (pre-reading skills), as well as the spelling and decoding processes, which are the foundations for fluency and reading comprehension.

This program occurs daily, consisting of a hybrid intervention, adopting virtual and real environments, respectively, a reading acquisition software (“I read,” four sessions per week, with a duration of 15 min each) and paper and pencil activities (one session per week, 45 min). Sessions occur in the school context, within school time, out of the classroom, within small groups (maximum 5 students). In Table 1 we present the paper and pencil activities organized in twenty sessions through purpose-built play-like materials.

**TABLE 1:** PPCL program: chronogram of activities, specifying specific goals.

**Session number**	**Month**	**Task name**	**Specific goal**
1st session	November	Mascot and teacher presentation + Snack time syllable	Presentation and Syllabic division
2nd session	November	I am a sound	Phonemic Awareness (initial phoneme) and Letter-Sound Knowledge
3th session	December	Sounds wheel	Phonemic Awareness (initial phoneme) and Letter-Sound Knowledge
4th session	December	Find the word	Phonemic Awareness (initial phoneme) and Letter-Sound Knowledge
5th session	January	Snack time—phoneme	Phonemic Awareness (phonemic segmentation)
6th session	January	Sound detective	Phonemic Awareness (segmentation and phonemic fusion)
7th session	January	Goldfish letters	Letter-Sound Knowledge
8th session	January	Bingo Diphthongs	Alphabetical decoding
9th session	February	Bingo CV	Alphabetical decoding
10th session	February	Dice	Alphabetical decoding
11th session	February	Words domino	Alphabetical decoding
12th session	March	Words factory	Alphabetical decoding
13th session	March	Bingo figure-word	Alphabetical decoding
14th session	March	Bingo—simple words	Alphabetical decoding
15th session	March	Words wheel (orthographic contextual rule: intervocalic < rr > and < r > for/R/and/r/)	Spelling decoding
16th session	April	Complete the words (orthographic contextual rule: intervocalic < rr > / < r > for/R/and/r/)	Spelling decoding
17th session	April	Complete the words (orthographic contextual rule: < ge/i > and < gue/i > ; for//and/g/)	Spelling decoding
18th session	May	Crosswords (orthographic contextual rule: < c > and < ç > for/s/)	Spelling decoding
19th session	May	Wheel and bingo words (orthographic contextual rule: < s > and < ss > for/z/and/s/)	Spelling decoding
20th session	May	Words Hopscotch	Spelling and Alphabetical decoding

Examples of the paper and pencil activities are: “I am a sound” or “Bingo.” The first activity (“I am a sound”) is intended to promote phonemic awareness: each student represents one sound. Student 1 represents the sound “rrrr”; Student 2 represents the sound “ffff”; Student 3 represents the sound “ssss” and finally, Student 4 represents the sound “zzzz.” Next, one card at a time (with the phoneme representation) is shown. The student’s task consists of verifying if the card starts with the sound they represent. The student representing the same sound as in the image is expected to raise her hand and say: “Sound!.” The game ends when all students accumulate six cards with the same sound.

In the “Bingo” activity, which aims to promote the decoding process, the instructor distributes one game board per student and asks each one to read every word on their board; then, the instructor gives a word card to one student at a time and asks them to read it. This procedure is followed by all students. Students are asked to look for the corresponding word on their board. That student who has the word on his/her board marks it with the card above it. The game ends when all students complete their board. Each bingo has different goals—alphabet decoding (using words with simple graphemes) or orthographic decoding (using words with complex graphemes) (For a detailed description and examples on the Portuguese orthography, cf Sucena et al., 2009; Sucena and Castro, 2011).

Along with these twenty sessions, the same competencies are promoted in a virtual environment, along with forty sessions adopting the “I read” software (Sucena et al., 2019). In Table 2 we present the virtual activities developed using the “I read” software. This is a free use software for all children included in the project. The “I read” is recently child-friendly computer software. It aims to develop and train foundation reading and spelling skills through systematic training and with different games. Activities are presented to the child with increasing difficulty: simple items first (regarding both syllabic structure and orthographic complexity) and complex items later. Children at risk of experiencing difficulties reading acquisition, at the beginning of their school path, or children with special educational needs can specifically benefit from this computer software.

**TABLE 2:** “I Read” tasks.

**General goals**	**Specific goals**	**Number of levels (planets)**	**Number of games (satellites)**
Simple vocalic grapheme	Phonemic awareness	1	3
	Letter-sound knowledge		
	Alphabetical decoding		
Oral diphthongs	Phonemic awareness	2	8
	Alphabetical decoding		
Nasal diphthongs	Phonemic awareness	1	4
	Alphabetical decoding		
Simple consonant grapheme	Phonemic awareness	2	63
	Letter-sound knowledge		
	Alphabetical spelling and decoding		
Complex consonant grapheme	Phonemic awareness	4	37
	Letter-sound knowledge		
	Orthographic spelling and decoding		

This study aims to present and evaluate the primary and main results of PPCL program implementation. A quasi-experimental design was adopted with two independent groups (intervention and comparative without intervention) and repeated measures (pre-and post-test).

## Materials and Methods

### Participants

Participants at pre-test included 311 Portuguese first graders, 173 boys (55.6%) and 138 girls (44.4%), between 5 years and 7 months and 7 years and 3 months (*M* = 6.17, *SD* = 0.83). Of these, 15 (2%) come from a minority ethnic group (Romani), and 33 (5%) have double nationality (born in Portugal but one foreign parent). For those with double nationality, it is important to emphasize that for 13 of them, the native language is Portuguese. The 311 participants were divided between the intervention group—206 (66.2%) attending 28 schools in the northwest of Portugal, and the comparative group—105 (33.8%) attending 20 schools in the northwest of Portugal. Participants were classified according to their socioeconomic background, 89 (43.2%) students from medium-high SES and 117 students (56.8%) from medium-low SES.

### Instruments

Demographic variables were collected with the collaboration of the school staff (e.g., age, sex). The alphabetic principle was assessed through phonemic awareness and letter-sound knowledge (pre-reading skills). Decoding and spelling (reading and spelling skills) were assessed through isolated word and pseudoword reading and spelling tasks.

Specifically, phonemic awareness was assessed with two tasks—onset awareness (ALEPE—Portuguese acronym for *Bateria de Avaliação da Leitura em Português Europeu*, in English—European Portuguese Reading Assessment battery, Sucena and Castro, 2011) and phonemic segmentation. The onset awareness was evaluated through the Subtest of Initial Phoneme Metalinguistic Phonological Awareness of ALEPE, which consists of identifying the common phoneme in a couple of words. This subtest contains three training items and twelve experimental items. Each item consists of a pair of words, with two syllabic structures, namely CV [Consonant-Vowel (open syllable) + CVC [Consonant-Vowel-Consonant (close syllable)]. The result is the total of correct answers. The critical value for the Subtest of Initial Phoneme Metalinguistic Phonological Awareness for the first grade is 80% (Sucena and Castro, 2011). Regarding Sucena and Castro (2011) a percentage of answers below the critical value alerts for an important gap in the performance of the assessed competence. This value was computed using a mean less than 1.5 Standard Deviation (Sucena and Castro, 2011). This is a common criterion adopted in the literature (e.g., Lezak et al., 2004). The phonemic segmentation was evaluated through one task that was built for that purpose, which consists of identifying the phonemes that constitute the word. The task is composed of two training items and five experimental items. Each item has the same syllable structure, namely VCV (Vowel-Consonant-Vowel). The result is the total of correct answers. There is no critical value for phonemic segmentation. Since there are no reference cutoff scores for Portuguese children on segmentation, the authors developed this task.

Letter-sound knowledge was evaluated through the Letters Reading Subtest and the Letters Spelling Subtest of ALEPE (Sucena and Castro, 2011). In the Letter Reading Subtest, the child is asked to read a list of letters (lowercase) displayed on the computer screen. This subtest consists of two training items and twenty-three experimental items. Both the sound and the letter name are accepted as correct answers. The training items are used only to guarantee that the child understands the task. The total result is the number of accurately identified letters. The critical value for first graders for reading letters is 87.4% (Sucena and Castro, 2011). In the Letter Spelling Subtest, the child is asked to spell letters dictated by the examiner (in lowercase format). This subtest consists of two training items and twenty-three experimental items. The total result equals the total number of accurately spelt letters. The critical value for first graders for writing letters is 93% (Sucena and Castro, 2011).

Word and pseudoword naming tasks of ALEPE (Sucena and Castro, 2011) were adopted to assess the decoding process. The child is required to read a list of words/pseudowords that are presented one by one on a computer screen, each for a maximum of 10 s. The stimuli list for first grade consists of four training items and eighteen experimental items. The items are composed of orthographically simple, consistent and inconsistent items for words and simple and consistent items for pseudowords. The critical value for the first grade is 57.5% for word naming and 50.8% for pseudowords. Cronbach’s alphas for the ALEPE words/pseudowords scales ranged between 0.46 to first graders and 0.72 to 2nd, 3^*rd*^, and 4th graders (Sucena and Castro, 2011).

Spelling was evaluated in two different tasks, one for words (*n* = 7) and the other for pseudowords (*n* = 5), each stimulus dictated by the examiner. There are orthographically simple, consistent and inconsistent items for words and simple and consistent items for pseudowords. The result is the total number of accurately spelt items. Since there are no cutoff scores for Portuguese children on spelling, the authors developed these exploratory tasks.

### Procedures of Data Collection

Authorizations were obtained from the school board and tutors. The objectives of the assessment were presented to both the school board and tutors, and the confidentiality of the data processing was guaranteed. Participants were administered the assessment tasks individually before and after the intervention, respectively, in September and in June 2019. The assessment team was constituted by eight reading and spelling training experts. The experts’ team also implemented the intervention program. All elements of the expert team worked in close collaboration, as well as with the principal researcher. The role of the expert team was to implement the intervention program previously designed. Participants allocated to the intervention program were selected by their teachers following two criteria: (i) being at risk of developing reading learning disabilities and (ii) not having any extra intervention regarding reading and spelling abilities. Participants were assigned to the comparative or the intervention group according to their performance on the pre-reading competencies—phonemic awareness and/or letter-sound knowledge. It was a deliberate option of the authors of this study to assign the students with the worst results to the intervention group. The cutoff point was determined at 1 Standard Deviation below the mean at (one or) the two tasks assessing phonemic awareness (and/or) letter-sound knowledge. Standards cutoffs are usually more conservative, ranging between 1.5 and 2 SD (Lezak et al., 2004). As the authors intended to identify at-risk children the option was to adopt a less conservative cutoff (1 SD), thus avoiding false negatives—to fail on identifying any at-risk children. Children in the comparative group followed the regular classes provided by the school teacher, whereas the intervention group benefited from the PPCL intervention along with the regular classes. There are no age differences between the intervention and the comparative groups. The proportion of students per SES was roughly equivalent: ca. 50% of the participants on both the intervention and the comparative group came from medium-high SES (specifically, 45 and 55%). Both groups were exposed to the (same) Portuguese educational program for reading acquisition, based on phonics instruction. After the intervention, the school teacher of the comparative group benefited from specific training on letter-sound, phonemic awareness, decoding and spelling strategies. Also, children allocated to the comparative were given free access to the “I Read” software.

### Data Analysis Procedures

Statistical analyses were performed through the *Statistical Package for the Social Sciences* (SPSS IBM) for *Windows*, version 25.0. A Two-Way MANOVA was conducted to analyze the group and time effect on the assessment dimensions. Before running this statistical test, we verified the fulfillment assumptions. The multivariate normality was not fulfilled. The homogeneity of variance-covariance matrices, verified by Levene and Box tests, was not fulfilled. The absence of uniqueness assumption was assured. The absence of multicollinearity is not fulfilled, since not all the correlations are less than 0.80 (Tabachnick and Fidell, 2013). The correlation between the letter spelling and the letter reading dimensions is 0.86. The sphericity assumption, verified by Bartlett’s test, was not fulfilled. The independence of the observations was ensured in the data collection procedures. To control for biases derived from the non-fulfilment Two-Way MANOVA assumptions, the value of Pillai’s Trace was considered (Tabachnick and Fidell, 2013). Cohen’s *d* was also calculated with the correction of Hedges and Olkin (1985), to evaluate the magnitude of the effect.

## Results

The inspection of the descriptive analyses reveals that at the pre-test, the comparative group performed better on pre-reading skills than the intervention group (around two times better); as for the reading skills the results are around zero for both the comparative group and the intervention group (cf. Table 3). At the post-test, the intervention group performed (i) better than the comparative group, both on pre-reading and reading skills, and (ii) above the critical value in all variables, whereas the comparative group scored below the critical value (in all variables).

**TABLE 3:** Effect of group and time in reading and writing letters, words and pseudo, onset awareness, and in phonemic segmentation.

			**Pre-test**				**Post-test**						**Group**	**Time**
			**Intervention group (*n* = 206) Mean (*SD*)**	**%**	**Comparative group (*n* = 104) Mean (*SD*)**	**%**	**Intervention group (*n* = 206) Mean (*SD*)**	**%**	**CV**	**Comparative group (*n* = 104) Mean (*SD*)**	**%**	**CV**	**Z (1,308)**	**Z (1,308)**
PRS	PA	OA^ a^	3.07 (3.68)	26%	5.74 (3.90)	48%	11.78 (0.99)	98%	>	9.06 (4.00)	76%	<	0.006	654.57***
		PS^*b*^	0.66 (1.19)	13%	1.63 (1.75)	33%	4.68 (0.87)	94%	–	2.85 (2.01)	57%	–	10.19**	759.36***
	LSK	Reading Letters^ c^	4.59 (3.47)	20%	11.25 (5.12)	49%	21.60 (2.02)	94%	>	17.35 (5.95)	75%	<	8.50**	2408.14***
		Writing Letters^*c*^	5.47 (3.71)	24%	11.05 (5.12)	48%	21.55 (2.66)	94%	>	18.03 (5.69)	78%	<	5.91*	2109.16***
RS	Decoding	Reading Words^*d*^	0.00	–	0.19 (0.88)	1%	10.67 (4.10)	59%	>	6.37 (5.05)	35%	<	57.43***	1003.63***
		Reading Pseudo^*e*^	0.00 (0.00)	–	0.16 (0.75)	1%	9.25 (3.79)	62%	>	5.04 (4.34)	34%	<	69.71***	867.67***
	Spelling	Writing Words^*f*^	0.00	–	0.00	–	3.10 (1.88)	44%	–	1.43 (1.60)	20%	–	60.13***	443.74***
		Writing Pseudo^*g*^	0.00	–	0.00	–	3.00 (1.45)	60%	–	1.43 (1.38)	29%	–	84.24***	671.16***

*PRS, Pre-reading skills; RS, reading skills; PA, Phonemic Awareness; LSK, Letter-Sound Knowledge; OA, Onset Awareness; PS, Phonemic Segmentation; M, Mean; SD, Standard Deviation.*

^*a*^Maximum correct answer 12; ^*b*^maximum correct answer 5; ^*c*^maximum correct answer 23; ^*d*^maximum correct answer 18; ^*e*^maximum correct answer 15; ^*f*^maximum correct answer 7; ^*g*^maximum correct answer 5; CV (Critical Value); < Below the critical value; > above the critical value; critical value for reading letters = 87.4; critical value for writing letters = 93; critical value for reading words = 57.5; critical value for reading pseudowords = 50.8; critical value for PA = 85; There is no critical value for spelling. ***p < 0.001 **p < 0.001 *p < 0.05.

The results of the Two-Way MANOVA indicate a statistically significant multivariate main effect for both time, *Pillai Trace* = 0.924, *Z*(8, 301) = 456.22, *p* < 0.001, partial eta squared = 0.924, and group, *Pillai Trace* = 0.428, *Z*(8, 301) = 28.17, *p* < 0.001, partial eta squared = 0.428, for all dimensions. There is also a multivariate main effect of the interaction between time and group in the evaluated dimensions, *Pillai Trace* = 0.712, *Z*(8, 301) = 92.89, *p* < 0.001, partial eta squared = 0.712.

Univariate tests show that the time factor has a statistically significant effect on pre-reading skills: Phonemic Awareness [onset awareness, *Z*(1, 1.0) = 654.57, *p* < 0.001, partial eta squared = 0.680, phonemic segmentation, *Z*(1, 1.0) = 759.36, *p* < 0.001, partial eta squared = 0.711], and on Letter-Sound Knowledge [reading letters *Z*(1, 1.0) = 2408.14, *p* < 0.001, partial eta squared = 0.887, writing letters *Z*(1, 1.0) = 2109.16, *p* < 0.001, partial eta squared = 0.873], as well as in reading skills: Decoding [(reading words *Z*(1, 1.0) = 1003.63, *p* < 0.001, partial eta squared = 0.765, reading pseudo *Z* 1, 1.0) = 867.67, *p* < 0.001, partial eta squared = 0.738], and on Spelling [writing words *Z*(1, 1.0) = 443.74, *p* < 0.001, partial eta squared = 0.590, writing pseudo *Z*(1, 1.0) = 671.16, *p* < 0.001, partial eta squared = 0.685]. Students have significantly higher values in these dimensions in the post-test as compared to the pre-test.

In addition, results indicate that the group factor has a statistically significant effect on pre-reading skills: [phonemic segmentation, *Z* 1, 308) = 10.19, *p* = 0.002, partial eta squared = 0.032] and Letter-Sound Knowledge [reading letters, *Z*(1, 308) = 8.50, *p* = 0.004, partial eta squared = 0.027, writing letters, *Z*(1, 308) = 5.91, *p* = 0.02, partial eta squared = 0.019], as well as on reading skills: Decoding [reading words, *Z*(1, 308) = 57.43, *p* < 0.001, partial eta squared = 0.157, reading pseudo, *Z*(1, 308) = 69.71, *p* < 0.001, partial eta squared = 0.185], and on Spelling [writing words, *Z*(1, 308) = 60.13, *p* < 0.001, partial eta squared = 0.163, writing pseudo, *Z*(1, 308) = 84.24, *p* < 0.001, partial eta squared = 0.215]. Students from the intervention group presented significantly higher values than those in the comparison group in the post-test. Comparison group students show significantly higher values than the ones in the intervention group on pre-test.

*Post hoc* tests indicate that the intervention group performed better than the comparative group on both pre-reading and reading skills; both groups revealed the same pattern of evolution, i.e., pre-reading as well as reading skills improved during the time, specifically in phonemic segmentation, Letter-Sound Knowledge (reading and writing letters), Decoding (reading words and pseudo), and Spelling (writing words and pseudo).

The magnitude of the effect between groups (*d* Cohen values) was analyzed with Hedges and Olkin correction (1985). *d* Cohen varies between 3.23 (phonemic segmentation) and 5.99 (reading letters) for the intervention group whereas in the comparative group varies between 0.70 (decoding) and 1.71 (reading letters).

## Discussion and Conclusion

This study sought to present the PPCL program and assess the primary and main results of its implementation. This program, like other international early reading interventions (e.g., Solheim et al., 2018; Jamshidifarsani et al., 2019), aims to promote pre-reading and reading skills with at-risk students, namely: knowledge of the alphabetic principle, through phonemic awareness and letter-sound knowledge, and the decoding and spelling processes, through word and pseudoword naming and spelling. A quasi-experimental design was adopted, with two independent groups (intervention and comparison without intervention) and repeated measures (pre-and post-test).

Both the intervention and the comparative group started 1st grade (pre-test) with low results (below 50%) on pre-reading skills, as would be predictable (Schaars et al., 2017). At the pre-test, both groups scored better on letter-sound knowledge and onset awareness tasks than on the remaining ones. Reading skills results were null as would be expected at the onset of first grade (Sucena and Castro, 2011). These results indicate some preparation for reading acquisition during preschool years, probably focusing on the knowledge of isolated letters. It seems that the students whose performance in letter spelling stands out have received training in letter spelling before entering first grade, although not so regarding phonemic awareness.

At the pre-test, the comparative group had significantly better results than the intervention group regarding pre-reading skills. Indeed, as previously noted it was the intention of the authors of this study to assign those students with the worst results to the intervention group. Once, due to the financial restrictions, it was not possible to include all children in the intervention program. As children in both groups started the school year with null or very low reading skills, it would be expectable to find a progression curve regarding those skills, which could be more or less pronounced according to variables such as language development, family risk of dyslexia, mother’s education, number of books at home, and/or family’s socioeconomic status (Pennington and Lefly, 2001; Bickford-Smith et al., 2005; Zalewska-Łunkiewicz et al., 2016; Porta and Ramirez, 2019; Raspin et al., 2019). The outcome expected at the end of the first grade is far more ambitious than performing significantly better than at the onset of the school year though. At the end of first grade, children are expected to have consolidated not only phonemic awareness and letter-sound knowledge but also to master the decoding process.

Indeed, at the post-test, the intervention group performed (i) above 50% in all dimensions (except for spelling words) and (ii) results are above the critical value in all dimensions, which indicates that these (once at risk) students are finishing the school year with satisfactory levels of reading skills (Kyle et al., 2013; Sucena et al., 2015).

The impact of the intervention was validated by three main results: (i) the intervention group started the school year with worse results than the comparative group and finished the school year with results significantly above of the comparative group, which has also been verified and reported by other authors (e.g., Nagler et al., 2015; Horne, 2017; Messer and Nash, 2017; Rosas et al., 2017), (ii) at the end of the first grade the intervention group performed above the critical values, in contrast to the comparative group whose results were below the critical values, and (iii) the magnitude of the effect is higher for the intervention group than for the comparative group. These results are consistent with those obtained with other programs that adopt a similar strategy and which have been applied in different schools in Portugal (e.g., Verdasca et al., 2019), as well as internationally (e.g., Ardoin et al., 2016; Hollands et al., 2016; Messer and Nash, 2017; Solheim et al., 2018; Jamshidifarsani et al., 2019; Martens et al., 2019; Volkmer et al., 2019). Even if those national programs do not use a quasi-experimental design with two independent groups (intervention and comparison without intervention) as this study use. By comparing pre-reading and reading skills between Portuguese children at risk of experience reading difficulties (subject and not subject to intervention), and by comparing their performance against 1st-grade critical values, we hope to have contributed to the first of many studies, assessing the impact of Portuguese reading promotion programs, and emphasizing the need to pursue scientifically informed strategies in reading intervention.

Despite the relevance of implementing an intervention program with a group of children at risk, it is worth mentioning one main limitation. The criteria for selecting the students for the intervention and the comparative group did not ensure equivalent groups and therefore the interpretation of the results should be taken with caution.

In future studies, it is important to guarantee that the selection criteria adopted ensure equivalent groups selection to be able to assess the effectiveness of the intervention. Even though it is important to highlight that a group with real and strong intervention needs did benefit from this intervention, with great educational and social implications.

Moreover, since at the post-test the comparison group scored below the critical value, it was the authors’ option to compensate the comparative group, for which teachers were invited to implement the PPCL in their classroom. In order to attain this goal school teachers of the comparative group have benefited from specific training on letter-sound, phonemic awareness, decoding and spelling strategies, accompanied by free access to the “I Read” software. Future research should assess the impact of maternal and/or paternal education on reading difficulties.

In conclusion, the PPCL methodology shows positive effects. It is expected that PPCL will help prevent early reading acquisition failure by promoting confident learners, willing to be fluent readers.

## Data Availability Statement

The raw data supporting the conclusions of this article will be made available by the authors, without undue reservation.

## Ethics Statement

Ethical review and approval was not required for the study on human participants in accordance with the local legislation and institutional requirements. Written informed consent to participate in this study was provided by the participants’ legal guardian/next of kin.

## Author Contributions

AS designed, supervised all the study, and also revised the article critically. CM analyzed the data and wrote the manuscript. AFS collected and insert the data on SPSS. All authors contributed to the article and approved the submitted version.

## Conflict of Interest

The authors declare that the research was conducted in the absence of any commercial or financial relationships that could be construed as a potential conflict of interest.
